# Determination of Fumonisins B1 and B2 in Food Matrices: Optimisation of a Liquid Chromatographic Method with Fluorescence Detection

**DOI:** 10.3390/toxins17080391

**Published:** 2025-08-05

**Authors:** Óscar Cebadero-Domínguez, Santiago Ruiz-Moyano, Alberto Martín, Elisabet Martín-Tornero

**Affiliations:** 1 Instituto Universitario de Investigación de Recursos Agrarios (INURA), Universidad de Extremadura, Avda. de la Investigación s/n, Campus Universitario, 06006 Badajoz, Spain; ocebadero@unex.es (Ó.C.-D.); srmsh@unex.es (S.R.-M.); 2 Departamento de Producción Animal y Ciencia de los Alimentos, Nutrición y Bromatología, Escuela de Ingenierías Agrarias, Universidad de Extremadura, Avda. Adolfo Suárez s/n, 06007 Badajoz, Spain; 3 Departamento de Química Analítica, Facultad de Ciencias, Universidad de Extremadura, Avda. de Elvas s/n, 06006 Badajoz, Spain

**Keywords:** mycotoxin, fumonisins, automatisation, HPLC-FLD, food matrices

## Abstract

Fumonisins, primarily produced by *Fusarium* spp. and *Aspergillus section nigri*, are common contaminants in maize, cereal grains, and other processed and derived products, representing a significant risk to food safety and public health. This study presents the development and optimisation of a high-performance liquid chromatography method with fluorescence detection (HPLC-FLD) for the quantification of fumonisin B1 (FB1) and B2 (FB2) in various food matrices. In contrast with conventional protocols employing potassium phosphate buffers as the mobile phase, the proposed method utilises formic acid, offering enhanced compatibility with liquid chromatography systems. An automated online precolumn derivatisation with o-phthaldialdehyde (OPA) was optimised through experimental design and response surface methodology, enabling baseline separation of FB1 and FB2 derivatives in less than 20 min. The method demonstrated high sensitivity, with limits of detection of 0.006 µg mL^−1^ for FB1 and 0.012 µg mL^−1^ for FB2, and excellent repeatability (intraday RSD values of 0.85% and 0.83%, respectively). Several solid-phase extraction (SPE) strategies were evaluated to enhance sample clean-up using a variety of food samples, including dried figs, raisins, dates, corn, cornmeal, wheat flour, and rice. FumoniStar Inmunoaffinity columns were the only clean-up method that provided optimal recoveries (70–120%) across all tested food matrices. However, the MultiSep™ 211 column yielded good recoveries for both fumonisins in dried figs and raisins. Additionally, the C18 cartridge achieved acceptable recoveries for both fumonisins in dried figs and wheat flour.

## 1. Introduction

Fumonisins are a group of mycotoxins primarily produced by the fungi *Fusarium verticillioides* and *Fusarium proliferatum*, which are commonly found in maize and other cereal grains. In addition, *Aspergillus* species, including *Aspergillus niger* and *Aspergillus welwitschiae*, are also capable of producing fumonisins, particularly fumonisin B2 (FB2). The most prevalent members of this group include fumonisin B1 (FB1), FB2, and B3 (FB3), with FB1 and FB2 being the most toxic and extensively studied. FB1 is a 2-amino-12,16-didmethyl-3,5,10,14,15-pentahydroxyeicosane esterified at C-14 and C-15 with propane-tricarboxylic acid, while FB2 is its 10-deoxy analogue [1]. The risk of food contamination with fumonisins is particularly high in countries where regulatory frameworks are absent, insufficient, or poorly enforced. This issue has become increasingly critical in regions such as Sub-Saharan Africa, South America, Latin America, and South/Southeast Asia, where the demand for maize and maize-based food products has risen substantially in recent years, heightening the risk of dietary exposure to these mycotoxins [2]. Exposure to fumonisins, particularly FB1, has been linked to a range of adverse health effects. Notably, FB1 has been classified as a Group 2B possible human carcinogen by the International Agency for Research on Cancer (IARC) [3]. Studies have shown that fumonisins can cause equine leukoencephalomalacia, porcine pulmonary edema, and non-genotoxic kidney or liver cancer in rats and mice [4,5,6,7]. Moreover, due to their significant health risks, including carcinogenic effects and potential links to esophageal cancer, the detection and quantification of fumonisins in food products are critical for ensuring food safety and protecting public health [8]. Effective detection methods for fumonisins are crucial due to the significant health risks associated with these toxins. To ensure food and crop safety, highly sensitive, selective, and robust analytical methods must be developed for reliable detection, precise monitoring, quality control, and comprehensive risk assessment of fumonisin [9]. Various methods are available for the determination of fumonisins in food matrices, including immunological techniques, chromatographic methods, and mass spectrometry-based approaches. Some of these methods are accompanied by various cleanup techniques aimed at extracting and purifying fumonisins present in food matrices. Some of these cleanup methods employ strong anion exchange (SAX) cartridges [10,11], immunoaffinity columns (IACs) [10,12], or RP-C18 solid-phase extraction (SPE) [1,13].

Although LC-MS methods have gained prominence in recent years, high-performance liquid chromatography (HPLC) coupled with fluorescence detection (HPLC-FLD) remains one of the most widely used techniques for determining fumonisins. This method is one of the most widely used methods for the determination of fumonisins in food matrices. It has demonstrated its ability to achieve low detection limits, making it well-suited for monitoring fumonisin levels in a variety of food products, such as corn and corn-based foods [14]. This method typically involves a pre-column derivatisation step using reagents such as o-phthaldialdehyde (OPA) or naphthalene-2,3-dicarboxaldehyde (NDA), which enhances the fluorescence properties of fumonisins, allowing for sensitive detection [15,16]. The derivatisation process is crucial as fumonisins lack inherent chromophores that would allow for direct detection via HPLC [16]. Numerous studies have validated the use of HPLC for analysing fumonisins. For instance, Ndube et al. [15] conducted a comparative study showcasing the efficacy of OPA and NDA for fluorescence detection in maize samples, highlighting the versatility of HPLC in mycotoxin analysis. Additionally, the method has been employed in various contexts, such as assessing the natural occurrence of fumonisins in corn products and evaluating exposure levels in specific populations [8,14]. The integration of HPLC with fluorescence detection not only provides a reliable analytical framework but also supports regulatory compliance and food safety monitoring efforts [17]. In conclusion, the determination of fumonisins using HPLC with fluorescence detection is a robust and sensitive approach that plays a vital role in ensuring food safety. The method’s ability to detect low levels of these mycotoxins in complex food matrices underscores its importance in public health and safety initiatives aimed at mitigating the risks associated with mycotoxin contamination.

Most studies employing HPLC-FLD methods for the determination of fumonisins have focused on corn and its derived products [10,18,19], typically using potassium phosphate as a buffering agent despite the risk of salt precipitation, which may lead to blockages and failures in analytical equipment. Although these mycotoxins can also be found in other grains, such as rice, wheat, and rye, as well as in dried fruits [20,21,22,23,24], research on these alternative matrices remains limited.

Thus, this study aimed to optimise an analytical method by replacing potassium phosphate with formic acid as the mobile phase modifier for the determination of FB1 and FB2 and to evaluate its effectiveness in detecting these mycotoxins in various food matrices (including dried figs, raisins, dates, corn, cornmeal, wheat flour, and rice). Additionally, different clean-up procedures were compared using a C18 cartridge, a Multisep^®^ 211 Fum cartridge, and the FumoniStar IAC.

## 2. Results and Discussion

### 2.1. Chromatographic Conditions Optimisation

During the development of this chromatographic method for fumonisin determination, particular attention was paid to the selection of the mobile phase. While most reported methods utilise potassium phosphate as a buffering agent [1], it is well-established that phosphate salts are susceptible to precipitation under certain conditions, particularly in the presence of organic solvents. These deposits can cause columns and tubing blockages, increased backpressure, and potential equipment damage, requiring frequent maintenance.

To mitigate these issues, we opted to replace potassium phosphate with formic acid as the mobile phase modifier. Formic acid is fully soluble in organic solvents such as methanol, which prevents precipitate formation and ensures cleaner and more stable operation. Additionally, formic acid facilitates better pH control in the acidic range, which is essential in fumonisin elution. The pH of the mobile phase was maintained at 3.3, as in the AOAC Official Method 2001.04 [25], but 1.5 mM formic acid was used for this instead of 0.1 M NaH_2_PO_4_. Methanol was selected as the organic solvent.

**Figure 4:**
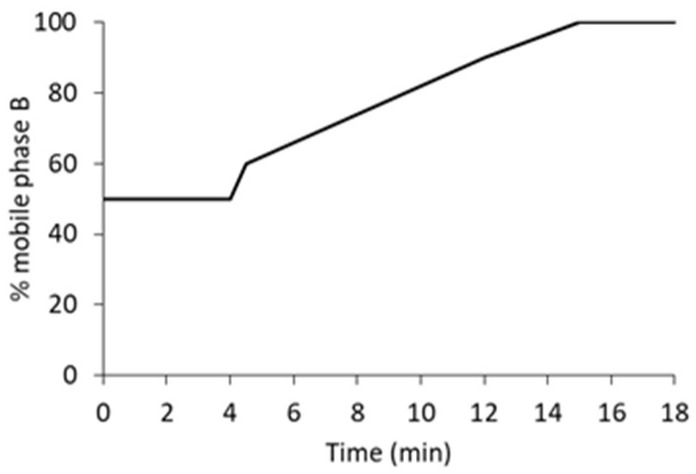
Gradient program optimised for the separation of fumonisin B1 and B2.

We evaluated various mobile phase compositions under isocratic conditions to identify the optimal proportion for analyte elution and resolution from the solvent front. The optimal separation from the front was achieved using a 50:50 (*v*/*v*) ratio of aqueous to organic phase under isocratic conditions. However, to attain baseline resolution of FB1 and FB2 and ensure adequate separation from residual derivatising reagent (OPA), a gradient elution program with increasing methanol concentration was necessary. The optimised gradient conditions are detailed in Section 4.3 and shown in Figure 4.

The temperature and pH of the mobile phase were both systematically evaluated and optimised. Elution of fumonisins was highly sensitive to pH fluctuations, with minor variations resulting in significant chromatographic changes. Several pH values, both above and below 3.3, were tested; however, none improved the resolution or sensitivity. Regarding column temperature, a range of 30 °C to 40 °C was explored. Increasing the temperature led to reductions in both retention time and analyte signal intensity. Based on these findings, a column temperature of 32 °C was selected as optimal.

**Figure 1:**
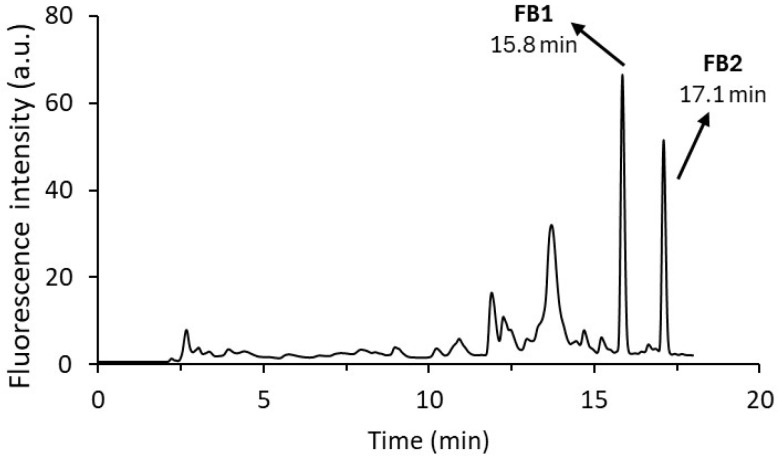
Chromatogram of the derivatives of fumonisins at a concentration of 1 µg mL^−1^ obtained under the optimised chromatographic method after online derivatisation with o-phthaldialdehyde (OPA).

The chromatogram obtained under these optimised conditions is shown in Figure 1.

### 2.2. Automatic Derivatisation Optimisation

After conducting a comprehensive literature review, it became clear that the most used derivatising agent for fumonisin determination is OPA in the presence of a thiol compound, such as 2-mercaptoethanol [15]. This derivatisation reaction occurs rapidly, yielding highly fluorescent isoindole derivatives. However, a notable limitation of this reaction is the instability of the resulting OPA–fumonisin adducts, which are prone to degradation over time due to hydrolysis and oxidation, particularly under exposure to light or prolonged exposure at room temperature [26]. Precise control of reaction time, temperature, mixing duration and intensity, and the waiting time before injecting the mixture is crucial to reducing variability and maintaining the stability of the derivative. These factors are difficult to control during offline (manual) derivatisation; therefore, online derivatisation has been proposed as a means to significantly improve precision.

In this study, all parameters involved in the online derivatisation process were optimised to develop an automated, robust method with maximum sensitivity and reproducibility. First of all, with all other parameters held constant, two aspiration protocols were evaluated to select the most effective configuration for derivatisation efficiency: sandwich injection (derivatising agent + sample + derivatising agent) and simple mixing (sample + derivatising agent). The results indicated that the sandwich injection yields the highest analytical response, which can be attributed to improved mixing efficiency between the sample and the derivatising agent, leading to enhanced derivatisation and signal intensity. Additional tests were conducted to evaluate the influence of the injection mode (draw sequentially vs. into a seat). Drawing the reagent sequentially provided the most effective derivatisation.

Following these preliminary studies, an experiment design was carried out to optimise the remaining parameters: volume of derivatising agent, mixing time, and pre-injection incubation time. A Box–Behnken design (BBD) was implemented involving these three factor variables, enabling the systematic evaluation of their linear, quadratic, and interaction effects on the chromatographic response (peak area). The tested variable ranges were as follows: derivatising reagent volume (4–14 µL), stirring (0–60 times), and pre-injection incubation time (0–5 min). A total of 15 experimental runs were performed using a standard mixture containing 5 µg mL^−1^ of fumonisins. All of the steps of the automatic injector were carried out at the default speed of 90 µL min^−1^. The optimum parameters were selected by employing a BBD.

Table 1 shows the results of ANOVA applied to assess the effects of the derivatising parameters studied and the statistical significance of the model for FB1 and FB2 determination via HPLC-FLD. To our knowledge, the impact of optimising derivatisation reagent quantity, its stirring, and pre-injection incubation time has not been previously evaluated. All three factors investigated demonstrated a significant (*p* ≤ 0.05) negative linear effect on the quantification of both fumonisins, with the derivatisation reagent concentration exerting the most substantial influence. Conversely, regarding the quadratic effects of the factors, only stirring and incubation time significantly (*p* ≤ 0.05) influenced the quantification of FB1, with no discernible impact observed on FB2. Additionally, significant interactions were observed for FB1 between agitation and incubation time (AB), and also between waiting time and the amount of derivatising reagent (BC), while no significant interactions were found for FB2.

**Figure 2:**
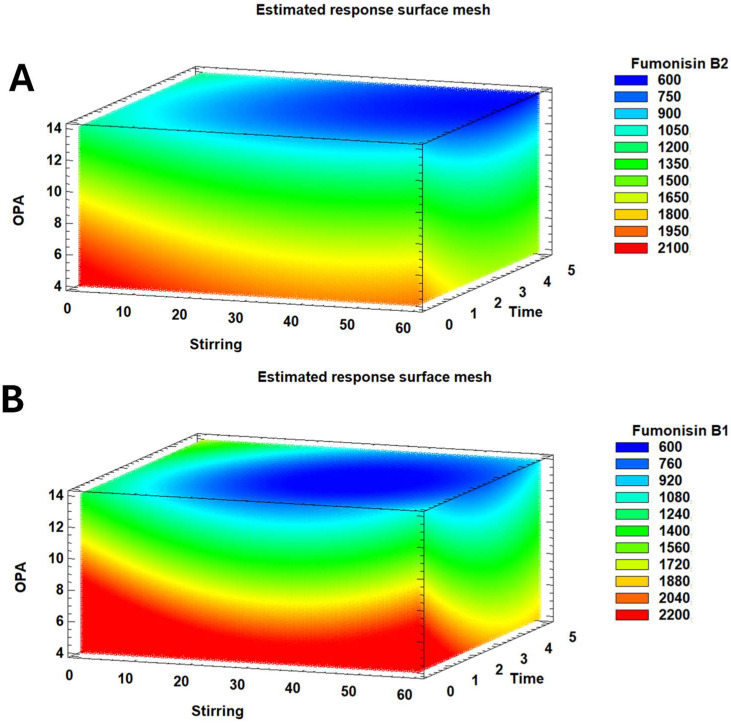
The response surface mesh illustrates the arbitrary unit area (UAA) of FB2 (**A**) and FB1 (**B**) as a function of the three derivatisation factors: stirring (times), incubation time after injection (min), and derivatisation reagent amount (OPA; µL). The colour gradient, from blue to red, signifies increasing fumonisin UAA.

Our findings indicate that the three analysed factors differentially influence the quantification sensitivity of each fumonisin. Figure 2 provides a detailed analysis of the complex relationships between stirring, OPA, and time about the estimated concentrations of fumonisin B1 and B2, expressed in arbitrary unit area (UAA). The colour gradients, ranging from deep blue to vibrant red, are essential for quantitative interpretation, with each shade mapped precisely to a specific concentration range, as indicated in the accompanying legends. For FB2, the scale ranges from 600 UAA (dark blue) to 2100 UAA (red), and for FB1, it ranges from 600 UAA to 2200 UAA. This visual encoding allows for immediate identification of regions yielding low (cooler colours) or high (warmer colours) fumonisin levels. A notable observation from Figure 2 is the general correlation between lower OPA values and increased concentrations of both fumonisins.

Specifically, the model for FB2 demonstrates that its quantification improves with decreasing agitation, incubation time, and derivatisation reagent amount (Figure 2A). In contrast, the model for FB1 is more complex, exhibiting significant linear and quadratic effects within the factors, as well as notable interactions among them. In this way, when the derivatisation reagent amount is minimised (4 µL), high sensitivity in FB1 quantification is achieved across a broad range of stirring and incubation time values (Figure 2B). Due to their quadratic impact, optimal quantification occurs at either lower or higher values of these latter two parameters.

The values of R^2^ (>0.988) and adjusted R^2^ statistics (>0.966) obtained for FB1 and FB2 represented a good fit with the model’s factors and ranges. The optimal conditions predicted for FB1 quantification were stirring 10 times, 1 min of incubation before injection, and 4.0 µL of derivatising reagent. For FB2, the optimal conditions for agitation and derivatisation concentration were the same. However, the optimal incubation time was longer at 5 min. Based on the statistical data of the model, the optimal parameters for automatic and online derivatisation of fumonisins were 2.0 µL of derivatising reagent (OPA) (4.0 µL in total, because it is injected in sandwich mode), 16.0 µL of sample, stirring 10 times, and 1 min of incubation before injection, as can be seen in Table 2.

### 2.3. Method Validation and Analytical Figures of Merit

The developed method was validated in terms of linearity, precision, low limit of detection (LOD), and low limit of quantification (LOQ). Linearity was evaluated by preparing standard solutions of both fumonisins as described in Section 4.4. Each standard was introduced into the chromatographic system and derivatised and eluted under optimised conditions. Calibration curves were built by plotting the peak area of the fumonisin derivatives against their respective concentration. The results are summarised in Table 3. As shown, excellent linearity was obtained for both analytes, with correlation coefficients (r^2^) exceeding 0.99.

The LOD and LOQ values were calculated using Equations (1) and (2). The LOD for FB1 was determined to be 0.006 µg mL^−1^, while that for FB2 was twice as high, at 0.012 µg mL^−1^. Previous HPLC-FLD methodologies for quantifying fumonisins exhibit slightly better LOD and LOQ values [1,10]; however, their use of a potassium phosphate mobile phase presents chromatographic limitations, as noted earlier. Nevertheless, the values obtained in this study are significantly lower than the permissible limits established by the European Commission, which include 800 μg kg^−1^ in cornflakes, 4000 μg kg^−1^ in unprocessed maize, 1000 μg kg^−1^ in maize intended for direct human consumption, 800 μg kg^−1^ in snacks and breakfast cereals, and 200 μg kg^−1^ processed maize-based foods and babies and young children foods [27].

Precision was evaluated based on both intraday and interday variability. Intraday precision (*n* = 10) and interday precision (evaluated over five consecutive days) were determined by repeated injections of a standard mixture (1 µg mL^−1^) of FB1 and FB2 under the optimised chromatographic conditions. The standard solutions were prepared daily by the same operator to ensure consistency and minimise variability. Precision was expressed as the relative standard deviation (RSD) of the peak areas. As shown in Table 3, intraday RSD values were 0.85% for FB1 and 0.83% for FB2. Interday RSD values were 2.4% and 5.9% for FB1 and FB2, respectively, indicating acceptable method robustness over time.

### 2.4. Application of the Method to Different Food Matrices

Direct injection of food samples was not feasible due to matrix interferences that compromise the accurate determination of fumonisins. Therefore, a sample clean-up step was necessary before chromatographic injection. According to the literature, most chromatographic methods incorporate SPE using C18 cartridges [1] or an IAC [28] to reduce matrix effects.

**Figure 3:**
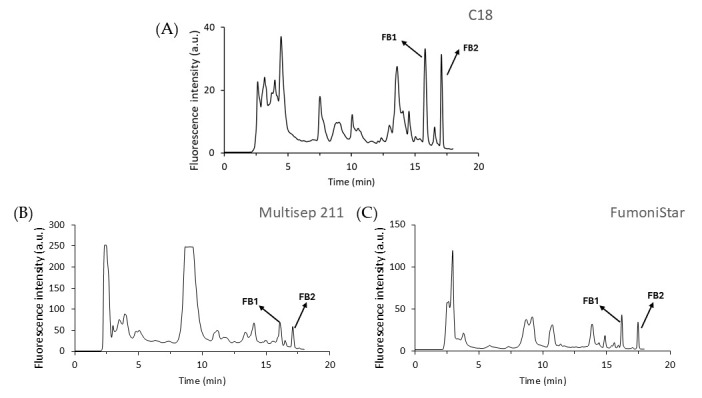
HPLC-FLD chromatograms of spiked dry figs cleaned with (**A**) C18 cartridge, (**B**) MultiSep 211 Fumonisins, and (**C**) FumoniStar Immunoaffinity Columns.

In this study, three types of SPE cartridges (C18, MultiSep 211, and the FumoniStar IAC) were evaluated for their effectiveness in removing matrix interferences across various food matrices. While the majority of published analytical methodologies for fumonisin determination focus on cereals and cereal-based products due to their high contamination risk, our study aimed to extend applicability to a broader range of matrices in which fumonisins have also been reported [21,22,29]. Therefore, the selected matrices included corn, cornmeal, wheat flour, rice, dried figs, raisins, and dates, chosen for their potential to be contaminated with fumonisins. Each sample was spiked with 1 µg mL^−1^ of FB1 and FB2 and subsequently subjected to extraction and clean-up procedures as described in Section 4.5. Unspiked samples were also analysed to assess background contamination, and no significant levels were detected in any of the analysed matrices. Each sample was analysed six times (*n* = 6). Figure 3 shows representative chromatograms of spiked dried figs following clean-up with each of the evaluated SPE methods. Moreover, the mean recovery rates of FB1 and FB2 for each cartridge–matrix combination are presented in Table 4.

According to the European Commission, acceptable recovery values for analytical methods detecting FB1 and FB2 in foodstuffs range between 70% and 120% [30]. Based on our results (Table 4), the FumoniStar IAC was the only clean-up method that fully complied with the European Union’s criteria across all tested food matrices except for dried figs. In this matrix, the recovery percentages for FB1 and FB2 were 60% and 65.2%, respectively, which is near the European Union’s minimum requirement [30]. The manufacturer’s specifications highlight the high performance of the IAC for cereals and their derivatives, particularly for those with established fumonisin legislative limits, such as maize and its by-products [27]. However, our findings suggest their broader applicability to complex matrices like dried fruits, including figs, dates, and raisins. Although no fumonisin limits are currently established for these products, the frequent presence of species from *Aspergillus* section *nigri* molds, which can potentially produce FB2, as well as this mycotoxin, indicates the potential relevance of such analysis [21,29,31,32]. This outcome is expected, as these cartridges employ specific antibodies that selectively bind to the target mycotoxins, enabling highly effective purification with excellent reliability, reproducibility, and average recovery rates. Nevertheless, the main disadvantages of the IAC are their high cost and single-use limitation [28,33].

In contrast, while C18 and MultiSep 211 cartridges did not meet the EU criteria in all matrices [30], they still provided satisfactory results in specific cases (Table 4). For instance, the MultiSep 211 consistently demonstrated good recovery values (70–120%) for both FB1 and FB2 in dried figs and raisins. The C18 cartridge also provided adequate recovery for both fumonisins in dried fig and wheat flour samples. This suggests that the more economical and readily available C18 cartridge is a suitable option for these specific matrices. Notably, in dried figs, both the C18 cartridge and MultiSep 211 extraction processes demonstrated significantly higher recovery efficiencies than the IAC (*p* ≤ 0.05). In contrast, both MultiSep 211 and C18 cartridges showed a low extraction efficiency for fumonisins in rice. The results for the C18 cartridge, concerning FB1 recovery, diverge from those reported by Hinojo et al. [20], who observed efficiencies approaching 100%. This discrepancy may be attributed to the previously demonstrated instability of FB1 and FB2 in rice flour, a phenomenon shown to be dependent on rice type [34]. The low recovery values observed for the other food matrices could be attributed to the interaction between FB and matrix macroconstituents, which form stable complexes. These interactions can significantly impact how well fumonisins are extracted from the matrix, with factors such as pH, temperature, and the amount of water playing key roles in achieving efficient recovery, parameters that would have less influence on the clean-up process with the IAC [35].

## 3. Conclusions

This study demonstrates that replacing the commonly used potassium phosphate with formic acid as the mobile phase in an HPLC-FLD method for the determination of fumonisins B1 and B2 in various food matrices enables significant optimisation of the analytical procedure. This mobile phase not only improved chromatographic performance but also proved to be more system-friendly by preventing salt precipitation. Nonetheless, maintaining strict pH control remains essential to achieve optimal chromatographic resolution and reproducibility. In addition, the optimised online derivatisation approach allowed precise control of reaction time, mixing duration and intensity, and incubation time, which enhanced the precision of the method. The experimental design used for derivatisation optimisation identified the amount of derivatisation reagent as the variable with the greatest influence on peak area. Optimisation efforts may focus on improving analytical parameters, particularly trace-level analysis in low-contamination samples. Regarding sample clean-up, three types of SPE columns were tested, with immunoaffinity cartridges providing acceptable recoveries across all tested samples. However, in certain matrices, more cost-effective alternatives such as Multisep or C18 columns also yielded satisfactory recoveries, supporting their potential use depending on the specific sample type. These findings contribute to the development of more robust and efficient analytical protocols for fumonisin determination and may support broader application in routine food safety monitoring and regulatory compliance.

## 4. Materials and Methods

### 4.1. Reagents and Standards

A crystalline solid FB1 (Cat# 62580) and FB2 (Cat# 13227) with purity ≥95% were purchased from Cayman Chemical. H_2_O and methanol were obtained from Panreac-Applichem (Barcelona, Spain) and Labkem (Barcelona, Spain), respectively. Acetonitrile (ACN) and C18 Sep-Pak SPE cartridges were purchased from Thermo Fisher Scientific (Madrid, Spain). o-phthaldialdehyde (OPA), 2-mercaptoethanol, and sodium tetraborate (Na_2_B_4_O_7_) were purchased from Sigma (Sigma-Aldrich, Madrid, Spain). All solvents were HPLC grade. Derivatisation reagent was prepared as follows (OPA): 40 mg OPA was mixed with 1 mL MeOH + 5 mL 0.1 Na_2_B_4_O_7_ + 50 µL 2-mercaptoethanol.

### 4.2. Precolumn Automatic Derivatisation Optimisation

FB1 and FB2 were chemically derivatised by using the injector program of the autosampler. The reagents were mixed by drawing them sequentially into the injection seat at the default speed (90 µL min^−1^). In brief, 2 µL of the derivatisation reagent was drawn, followed by 16 µL of the sample vial and another 2 µL of the derivatisation reagent. The mix in the sample loop was mixed 10 times, and after 1 min, it was injected. Moreover, between each drawing step, the needle was washed with methanol. Table 2 presented above in the Results and Discussion section (Section 2.2) summarises the steps of the automatic injection. A BBD with three factors was employed to model the combined effects of the reaction parameters (amount of derivatising reagent, number of stirring cycles, and incubation time) in the OPA derivatisation process. The variable factor conditions are shown in Table 5.

### 4.3. HPLC-FLD Analysis

For HPLC analysis, an Agilent Technologies 1260 Infinity II High Performance Liquid Chromatograph was used. The system was equipped with an online degasser, quaternary pump (G7111B), autosampler (G7111B), column oven compartment (G7129A), UV-Vis diode array detector (G7117C), and fast scanning fluorescence detector (G7121B). ChemStation software was used to control the instrument, data acquisition, and data analysis.

Derivatives separation was carried out on a Raptor C18 column (150 × 4.6 mm; 2.7 μm; Restek, Bellefonte, Pennsylvania, USA) with its appropriate guard column. The column oven was set at 32 °C. The mobile phase solvents were 1.5 mM formic acid (pH 3.3) (A) and methanol (B), both filtered through a 0.22 μm membrane filter. The gradient program was as follows: 50% of mobile phase B was maintained for 4 min, followed by three linear gradients: from 50 to 60% B between 4.0 to 4.5 min, from 60% to 90% B between 4.5 to 12.0 min, and from 90% to 100% B between 12 to 15 min (Figure 4). Mobile phase B (100%) was held until 18.0 min. After each analysis, a 7 min post-run was used to return to the initial mobile phase composition. The flow rate was set at 0.5 mL min^−1^, and the injection volume was 20 μL.

FLD detection was performed at 335 nm of excitation and 440 nm of emission wavelengths.

### 4.4. Calibration Curve and Figures of Merit

Stock standards solutions of FB1 (100 µg mL^−1^) and FB2 (50 µg mL^−1^) were prepared in ACN/water (50:50, *v*:*v*) and stored at 5 °C. The calibration curve was constructed by analysing working solution mixtures at five concentration levels with concentrations ranging from 0.05 to 5 µg mL^−1^. Three replicates of each concentration level were analysed under optimised HPLC conditions, and all of them were previously filtered through a 0.22 μm PTFE filter. Calibration curves were built by plotting the peak area versus the standard concentration.

The limits of detection (*LOD*) and quantification (*LOQ*) were estimated based on the standard deviation of the response for low concentration replicates (*σ*) and the slope of the calibration curve (*m*), as recommended by ICH guidelines, using the following equations:

(1) $$LOD=3.3*\;\frac{\sigma }{m}$$

(2) $$LOQ=10*\;\frac{\sigma }{m}$$

Precision was determined as the percentage of the relative standard deviation of both intraday and interday repeatability (*n* = 5), using a mixed standard solution of the two fumonisins at a concentration of 1 µg mL^−1^.

### 4.5. Food Matrices Preparation and Clean-Up Procedures

Seven different food matrix samples were used, including dry figs, raisins, dates, corn, corn, cornmeal, wheat flour, and rice. All products were purchased from local supermarkets. The samples were homogenised at 650 W in a chopper (JATA PC123N). Six independent replicates were carried out for each food matrix.

During the experiment, 0.3 g food matrices were spiked with fumonisins at a concentration level of 1 µg ml^−1^ and extracted with ACN/water (1:1 *v*/*v*), shaken, and centrifuged before the application of different clean-up methods: C18 cartridge, Multisep^®^ 211 Fum cartridge, and the FumoniStar IAC.

(A) C18 cartridge (Thermo Fisher Scientific, Waltham, MA, USA): The supernatant (2 mL) was collected and diluted with 5 mL of water. Samples were loaded onto a C18 cartridge, which was preconditioned with 5 mL MeOH and 5 mL water. After washing with 5 mL water and 2 mL ACN:H_2_O (1:9 *v*/*v*), fumonisins were eluted with 2 mL ACN:H_2_O (1:1 *v*/*v*). Samples were filtered through a 0.22 μm PTFE filter and stored at 4 °C until FB1 and FB2 analysis (<12 h).
(B) MultiSep 211 Fumonisins (Romer Labs, Getzersdorf, Austria): The supernatant was filtered, and the pH was adjusted to a range of 6–9. Next, 3 mL extract was mixed with 8 mL of MeOH: H_2_O (3:1 *v*/*v*) and applied to the MultiSep 211 column, previously prewashed with 5 mL of MeOH followed by 5 mL of MeOH: H_2_O (3:1 *v*/*v*). After the sample passed through the cartridge, the column was washed with 8 mL of MeOH:H_2_O (3:1, *v*/*v*), followed by 3 mL of MeOH. Fumonisins were eluted with 10 mL of ACN/water (1:1 *v*/*v*) and evaporated to dryness with a Rotavapor. The resulting residue was dissolved in 0.5 mL of ACN:H_2_O (1:1 *v*/*v*) and stored at 4 °C until its HPLC analysis (<12 h).
(C) FumoniStar IAC (Romer Labs): First, 1 mL of the extracted sample was added to 4 mL of PBS, mixed, and filtered. Then, 1 mL of the diluted extract was passed through an IAC and washed with 1 mL of PBS at an approximate flow rate of 1 mL/min. Fumonisins were eluted using 3 mL of ACN:H_2_O (1:1, *v*/*v*). The eluate was evaporated and redissolved in 0.5 mL of ACN:H_2_O (1:1, *v*/*v*). Samples were filtered through a 0.22 μm PTFE filter and stored at 4 °C until FB1 and FB2 analyses (<12 h).

### 4.6. Statistical Analysis

To model the amount of OPA reagent, the number of stirring cycles, and the incubation time variables (Table 5) on the derivatisation process, a 3-factor, 3-level Box–Behnken design combined with surface response methodology was applied. Surface response methodology was performed by employing StatGraphics Centurion XVI version 8.0 software. The quadratic model was as follows:

$$Y=\beta_{0}+\beta_{1}X_{1}+\beta_{2}X_{2}+\beta_{3}X_{3}+\beta_{12}X_{1}X_{2}+\beta_{13}X_{1}X_{3}+\beta_{23}X_{2}X_{3}+\beta_{11}X_{1}^{2}+\beta_{22}X_{2}^{2}+\beta_{33}X_{3}^{2}$$

where *Y* is the response variable (FB1 or FB2 concentration) predicted by the model; *β*0 is an offset value; *β1, β2, and β3* are the regression coefficients for the main (linear) terms; *β11, β22*, and *β33* are quadratic effects; *β12, β13, and β23* are interaction effects; and *X1, X2, and X3* are the independent variables. The model was used to estimate the FB1 or FB2 concentration at different amounts of OPA reagent, number of stirring cycles, and incubation time (Table 5). The software also generated an ANOVA, establishing statistical significance at the 95% confidence level. Optimal levels for the FB1 and FB2 concentrations under each variable analysed were also obtained with the same statistical program.

Finally, statistical analysis of the data from fumonisin recoveries from food matrices was carried out using SPSS for Windows, version 21.0 (IBM Corp, Armonk, NY, USA). The differences between groups were studied via one-way ANOVA and separated with Tukey’s honest significant differences test (*p* ≤ 0.05).

## Figures and Tables

**Table 1:** ANOVA results for the response surface quadratic model and optimal derivatising parameters (OPA amount, stirring times, and incubation times) for fumonisin determination via HPLC-FLD.

ANOVA	FB1		FB2	
	*p*-Values	Reg. Coeff. ^1^	*p*-Values	Reg. Coeff.
Constant		3514.3		2386.9
A: Stirring	**0.023 ^2^**	−29.8	**0.016**	−12.4
B: Incubation Time	**0.002**	−349.3	**0.044**	−127.7
C: OPA	**0.000**	−144.7	**0.000**	−34.4
AA	**0.000**	0.4	0.075	0.1
AB	**0.027**	−1.6	0.276	−0.9
AC	0.803	0.1	0.978	0.0
BB	**0.001**	46.7	0.070	22.0
BC	**0.004**	13.2	0.796	1.0
CC	0.439	−0.9	0.056	−3.8
*R-squared statistics*				
R^2^	0.996		0.988	
R^2^ (adjusted by g.l.)	0.989		0.966	
*Optimal SFE conditions*				
Stirring (times)	10		10	
Time (min)	1		5	
Derivatasing reagent (OPA; µL)	4		4	

^1^ Reg. Coeff.: regression coefficients; ^2^ *p*-values lower than 0.05 are statistically significant (bold font).

**Table 2:** Automatic injection steps for the online pre-column derivatisation of FB1 and FB2.

Function	Quantity (Volume or Times)	Compound
Draw	2 µL ^1^	Derivatisation reagent
Needle wash	2 times	MeOH
Draw	16 µL	Sample
Needle wash	2 times	MeOH
Draw	2 µL ^1^	Derivatisation reagent
Needle wash	2 times	MeOH
Mix	10 times ^1^	Air
Incubation	1 min ^1^	
Inject	20 µL	Reaction mixture

^1^ Optimal parameters modelling for automatic and online derivatisation.

**Table 3:** Analytical parameters for the optimised chromatographic method.

	FB1	FB2
Linear range (µg mL^−1^)	0.05–5.0	0.05–5.0
Regression eq. (SD)	y = 528 (4) x – 12 (1)	y = 410 (5) x + 10 (2)
Determination Coefficient (r^2^)	0.9993	0.9987
LOD (µg mL^−1^)	0.006	0.012
LOQ (µg mL^−1^)	0.017	0.038
Intraday repeatability (RSD %)	0.85	0.83
Interday repeatability (RSD %)	2.4	5.9

**Table 4:** Fumonisin recoveries in various matrices spiked with FB1 and FB2, separated by different columns.

Recovery (%)	C18 Cartridge		MultiSep 211 Fumonisins		FumoniStar Immunoaffinity Columns	
	FB1	FB2	FB1	FB2	FB1	FB2
Dried figs	89.9 ^b^* ± 7.4	72.2 ^2^ ± 2.3	115.9 ^c^ ± 3.1	105.4 ^3^ ± 2.8	60.0 ^a^ ± 2.2	65.2 ^1^ ± 6.2
Raisins	38.9 ^a^ ± 2.3	<20 ^1^	124.5 ^c^ ± 6.2	77.9 ^2^ ± 1.5	72.6 ^b^ ± 4.9	86.1 ^3^ ± 5.5
Dates	<20 ^a^	49.7 ^2^ ± 5.5	<20 ^a^	<20 ^1^	81.3 ^b^ ± 0.6	92.5 ^3^ ± 6.2
Corn	<20 ^a^	77.5 ^2^ ± 8.5	125.6 ^c^ ± 4.9	<20 ^1^	97.3 ^b^ ± 3.1	99.6 ^3^ ± 1.4
Cornmeal	35.2 ^a^ ± 7.6	83.9 ^2^ ± 0.3	118.2 ^c^ ± 8.6	<20 ^1^	87.4 ^b^ ± 8.8	101.4 ^3^ ± 9.8
Wheat flour	66.5 ^b^ ± 4.3	78.6 ± 3.8 ^2^	<20 ^a^	<20 ^1^	75.2 ^c^ ± 6.4	83.7 ^2^ ± 4.7
Rice	<20 ^a^	<20 ^1^	<20 ^a^	<20 ^1^	74.4 ^b^ ± 3.8	85.1 ^b^ ± 8.7

* For a given row, values with different superscript letters indicate a statistically significant difference (*p* ≤ 0.05) in the recovery efficiency of FB1. Similarly, different superscript numbers denote a statistically significant difference (*p* ≤ 0.05) in the recovery efficiency of FB2.

**Table 5:** Modelling derivatisation: reagent amount, stirring, and incubation conditions.

Block	Stirring Cycles	Incubation Time(s)	OPA Reagent (µL)
1	60	1	9
2	10	3	14
3	10	1	9
4	35	1	4
5	60	3	4
6	10	5	9
7	60	5	9
8	35	5	14
9	35	5	4
10	10	3	4
11	60	3	14
12	35	1	14
13	35	3	9
14	35	3	9
15	35	3	9

## Data Availability

The original contributions presented in this study are included in the article. Further inquiries can be directed to the corresponding author.

## Abbreviations

The following abbreviations are used in this manuscript:

FB	Fumonisin
OPA	o-phthaldialdehyde
SPE	Solid-phase extraction
IAC	Immunoaffinity columns
BBD	Box–Behnken design
